# The current status of nurses–doctors collaboration in clinical decision and its outcome in Tanzania

**DOI:** 10.1002/nop2.360

**Published:** 2019-08-21

**Authors:** Joanes Faustine Mboineki, Changying Chen, Dolla Deo Gerald, Cecilia Amponsem Boateng

**Affiliations:** ^1^ School of Nursing Zhengzhou University Zhengzhou China; ^2^ School of Nursing and Public Health, College of Health Sciences The University of Dodoma Dodoma Tanzania; ^3^ Mbweni Hospital Dar es Salaam Tanzania; ^4^ School of Public Health Zhengzhou University Zhengzhou China

**Keywords:** clinical decision‐making, collaboration, doctors, image of nursing, nurses, nursing, perception

## Abstract

**Aim:**

The aim of this study was to establish the current level of collaboration between nurses and medical doctors (MDs) in the making of clinical decisions.

**Design:**

Descriptive qualitative design was applied in this study.

**Methods:**

Semi‐structured interviews were conducted to collect qualitative data. Contents were arranged according to their similarities, whereas content analysis was used to identify explanatory themes.

**Results:**

Nurses feel disrespected when medical doctors (MDs) ignore their opinions. The impression of lower level of education of nurses is seen as a cause to their opinions been ignored by the medical doctors. Nurses sometimes adhere to the instructions of MDs, but on other times, they carry on with their own proposed treatment.

**Implications for nursing practices:**

Involvement of nurses in clinical decisions will enable nurses to effectively advocate for patients.

## 1 INTRODUCTION

Collaboration is essential in clinical practice; particularly, it increases health workers motivation, reduces treatment errors, increases effective care and reduces the weakness of professional performance (Foth, Block, Stamer, & Schmacke, 2015; Zamanzadeh, Irajpour, Valizadeh, & Shohani, 2014). Generally, collaboration is defined as working with other persons to accomplish the targeted goal/s (Zamanzadeh et al., 2014). Collaborative approaches differ depending on discipline, but each approach is result oriented. Patient's recovery depends on collaboration between different healthcare providers, and such medical collaborations help to bring solutions in situations requiring complex treatment (Green & Johnson, 2015). Without collaboration of healthcare professionals, it is impossible to meet all of the patient's demands (Mahdizadeh, Heydari, & Moonaghi, 2017).

Collaboration between nurses and medical doctors (MDs) in clinical facilities is a kind of teamwork, which is reported to result into quality healthcare services. On the other hand, lack of collaboration between nurses and MDs increases medical errors and also leads to suboptimal healthcare services (Elsous, Radwan, & Mohsen, 2017); collaboration between nurses and MDs should, therefore, focus on respect, good communication and shared clinical decision power (Elsous et al., 2017).

Although collaboration is essential to clinical facilities, nurses in Tanzania complain of lack of respect from MDs and the absence of shared clinical decision‐making power (Langway, 2017). Meanwhile, the guiding principle of medical ethics requires MDs at all levels to recognize and respect the expertise of other health workers. Additionally, the same principle requires MDs to collaborate with other health workers in the interest of providing the best possible holistic healthcare services (MAT, 1995). Inarguably, teamwork is more significant than skills and knowledge in the provision of quality healthcare services (Ishijima, Eliakimu, Takahashi, & Miyamoto, 2014).

A clinical decision is defined as the process that starts from assessing patients and diagnosis and ends up with the decision of what is to be done after the assessment and diagnosis is conducted (Catarina & Campos, 2009). However, MDs are not ready to listen to and accept the modes of treatments of patients as proposed by nurses (Johnson, 2009). It is, moreover, argued that nurses should be involved in shared clinical decision power for patient's treatments since they spend more time with patients and tend to understand the needs of patients better than MDs (Mcclelland, Switzer, & Pilcher, 2012).

Literature about nurse–MDs collaboration in Tanzania is limited. The few that have been conducted rather reported the distresses among nurses due to lack of respect from other healthcare professionals (Häggström, Mbusa, & Wadensten, 2008). This study, therefore, intends to establish the nature and level of collaboration between nurses and MDs in the making of clinical decisions in Tanzania. Findings from this study could be factored into strategies for improving healthcare delivery in Tanzania.

### 1.1 Nurses in Tanzania

In the Tanzanian perspective, nurses are defined as authorized licensed professionals with adequate knowledge and competency to carry out quality nursing care to individuals, families and communities, and they are categorized according to their different academic training levels such as certificate, diploma, advanced diploma, bachelor degree, master degree and doctorate degree (TNMC, 2014). The curricula for training nurses include both theory classes and clinical practices. Besides, they are also trained to provide holistic nursing care in aspects of culture and spiritual care (Tjoflåt et al., 2018).

Tanzania has a national population of 47,524,276. There are 31,618 nurses and midwives with different levels of the above‐mentioned certificates. Approximately, there is a one nurse or midwife ratio per 1,374 populations, as against the World Health Organization (WHO) recommendation of one nurse/midwife per 492 populations (Tarimo, Moyo, Masenga, Magesa, & Mzava, 2018).

All nursing activities in Tanzania including registering and enrolment of nurses, establishing standards of proficiency and issuing licence to the nurses are regulated by the Tanzania nursing and midwifery council (TNMC) (TNMC, 2010). This council has established principles that govern the practice of nursing and ensure the provision of quality care to patients.

Nurses are also trained to work as members of the multidisciplinary team to protect the interest of clients (TNMC, 2014). A nurse, therefore, can prescribe medicine and perform minor surgeries and other complex tasks if only he or she possesses the knowledge and in the absence of an MD (TNMC, 2014). The code of ethics and professional conducts for nurses in Tanzania emphasizes that nurses must maintain professional competence and collaborate with other healthcare providers as a team (TNMC, 2015).

### 1.2 Medical doctors (MDs) in Tanzania

In 2012, the doctor to population in Tanzania ratio was 0.3 per 10,000 (Sirili et al., 2019). Recently, these statistics have changed to one medical doctor per 20,010 people, regardless of the WHO recommendation of one medical doctor to 4,000 people (Tarimo et al., 2018). In postindependence, the country introduced subcadres within the profession with the aim of carrying out task‐shifting roles for doctors to aid the shortage of MDs within the country. These included Assistant Medical Officers (AMOs) and Clinical Officers (COs) (Sirili et al., 2019). In 2015, the country had 11 medical schools producing students with diploma, advanced diploma and degree programme (Sirili et al., 2019).

## 2 METHODOLOGY

### 2.1 Design

A descriptive qualitative study was conducted to obtain cases deemed rich in information (Lambert & Lambert, 2012). The study purposively sampled nurses and doctors in Tanzania. Each was asked to respond to their lived clinical experiences about collaboration between nurses and doctors.

### 2.2 Setting and sampling technique

The study was conducted in five hospitals located in Dar es Salaam, Tanzania. Two were public hospitals ranked as referral hospitals, and three private hospitals. Nurses and medical doctors (MDs) recruited into the study had either certificate, diploma or degree from recognized training schools. Purposive sampling method was applied in recruiting the participants. Frequent and similar responses from different healthcare providers indicated that the information was saturated.

### 2.3 Ethical consideration

The research proposal was submitted for ethical clearance to the ethical committee at University of Dodoma. It was confirmed to have no harm to healthcare providers. Informed consent was obtained from all participants, and information collected from healthcare providers was kept confidential.

### 2.4 Data collection procedures

The data were collected from October–November 2018. Semi‐structured interviews particularly face‐to‐face interviews were conducted to investigate their lived clinical experiences on nurse–doctor collaboration in decision‐making. Both Principal Investigator and research assistants were native Tanzanians who were able to speak Swahili and English to facilitate the processes of the interview. The interviews of nurses and MDs were carried out using a similar interview guide with open‐ended questions. The interview guide was prepared in English and translated into native Swahili language for the convenience of participants. The interview guide had several questions: part 1 looked into the experiences on the relationship of nurse–doctor collaboration in making decisions on patient's treatments, while part 2 focused on the current influence of the relationship of nurses and doctors in clinical decisions in the provision of healthcare services. A digital audio recorder was used to record the interview conversations. Prior to the interview day, all healthcare providers were contacted and informed about the research work and their participation in the study. Healthcare providers and researchers agreed on the interview day, time and place. A reminder text message was sent to each participant one day before the interview. The interviews were conducted in quiet places to avoid distractions and other interferences (Tjoflåt et al., 2018). Each interview was started by introduction to establish trust from participant. The interviews were not hastily performed for participants to have time to share their experiences and perceptions.

### 2.5 Data analysis

The analysis started with transcription (Bailey, 2008). All audio‐recorded data were converted into written Swahili. The Principal Investigator and his assistant did transcription separately and later exchanged the transcripts to make comparison for the identification of missing information. Transcripts were later translated into the English language by the cooperation of both researchers (Smith, Chen, & Liu, 2008). Contents were arranged according to their similarities, and later, content analysis was used to identify explanatory themes (Graneheim & Lundman, 2004).

## 3 RESULTS

### 3.1 Sample characteristics

Twelve healthcare providers dominated by nurses and MDs participated in the study. Most of them were females. The demographic characteristics are summarized in Table 1.

**Table 1:** Particulars for healthcare providers

Participants (p)	Sex	Working place	Age	Education level	Profession	Cadre	Department
P1	Female	Private hospital	27	Diploma	Nurse	Registered nurse (RN)	Maternity ward
P2	Male	Private hospital	28	Diploma	Nurse	Registered nurse (RN)	General ward
P3	Male	Private hospital	25	Certificate	Nurse	Enrolled nurse (EN)	General ward
P4	Female	Private hospital	28	Diploma	Nurse	Registered nurse (RN)	Maternity ward
P5	Female	Private hospital	40	Certificate	Nurse	Enrolled nurse (EN)	Maternity ward
P6	Male	Private hospital	32	Bachelor	Doctor	Medical doctor (MD)	Maternity ward
P7	Female	Public hospital	26	Diploma	Nurse	Registered nurse (RN)	Maternity ward
P8	Female	Public hospital	24	Diploma	Nurse	Registered nurse (RN)	Maternity ward
P9	Female	Public hospital	32	Bachelor	Doctor	Medical doctor (MD)	Male medical ward
P10	Female	Private hospital	29	Bachelor	Doctor	Medical doctor (MD)	Male surgical ward
P11	Male	Public hospital	40	Bachelor	Doctor	Medical doctor (MD)	Paediatric ward
P12	Male	Private hospital	30	Bachelor	Doctor	Medical doctor (MD)	Male medical ward

### 3.2 Overview

Three major themes and sixteen subthemes were identified from this study: (a) Actions taken by nurses when MDs reject their opinion, (b) Factors influencing MDs in the rejection of nurse's advice and (c) The impact of doctors' rejection on nurse's advice. The abbreviation “P” is frequently used in this section of the result to signify the “participant”. Refer Table 2

**Table 2:** Summary of themes and subthemes

Themes	Subthemes	Reasons
1. Actions taken by nurses when MDs reject their opinion	Take no further actions	They believe MDs own all decision authority for patient treatment
	Do not follow MD's orders	They think MD's orders will not improve patient's conditions; therefore, they decide to implement their own treatment plan
	Consult fellow nurses	They explore if their opinions are right and if found so they all join hands to confront MDs
	Present books to MDs as references	To prove that what they speak is evidence‐based
	Report MDs to the hospital's management	They trust the administration would provide punishment to denounce the MDs behaviour
2. Factors influencing MDs to reject nurse's advice	Lack of respect	MDs feel nurses do not deserve to have shared clinical decision power
	Education background	MDs perceive they are well educated than nurses
	Daily clinical performances of a nurse	If the clinical performance of nurse is perceived poor, their opinion is likely to be rejected
	MDs want to keep their respect	MDs perceive clinical decision power is their opportunity to keep their professional status
	Overworking	Shortage of MDs workforce creates overwhelming due to many patients that reduces MDs attention to nurses' opinions
	Nurses opinion not based on scientific facts	Doctors provide treatment from evidence based and not relying on individual experiences
3. Impacts of doctor‐rejecting nurse's advice	Lose job morale emotionally stressed	They feel they are not part of healthcare team because their contributions are not considered, and hence, patients are not adequately advocated
	Diminishing of a relationship between these professionals	The rejection results into disunity because nurses perceive MDs disrespect them
	Nurses feels inferior	The confidence of nurses in providing care is diminished and feels inferior because they do not have power to advocate the patients
	Effect to patients	Patient may receive suboptimal treatment

### 3.3 Actions taken by nurses when MDs reject their opinion

Different experiences were identified from healthcare providers on how nurses respond when their suggestions concerning patient's treatments are not taken into consideration. Nurses are reported to have confidence in making suggestions to MDs; however, when these are ignored, nurses take no further actions but are left with reserved feelings that MDs have the overall authority for patient's treatment**:**

When MDs show no interest to my suggestions, i remain quiet and I don't report anywhere or take any action. (P2, RN)

I make sure I speak what I think will help the patient to recover and if an MD reject my opinion then I comply to his decision because has authority. (P5, EN)

Conversely, most nurses expressed that they do not follow MD's orders after their suggestions concerning patient's treatments are not taken into consideration:

When I realize that an MD has deliberately rejected my right opinion without a genuine reason, I choose not to follow his orders and rather I implement what I believe is the best treatment for the patient. (P1, RN)

### 3.4 Example of a clinical case reported by the participant

One day a patient came to our hospital with an asthmatic problem. I advised the MD that we should have cannulated the patient and administered hydrocortisone and aminophylline, the MD despised my opinion and decided to give another order. After I discussed with other nurses within the department, they found that i had the right opinion, we decided to give hydrocortisone and aminophylline. The patient condition improved and was restored to normal. (P3, EN)

Evidently, most nurses soon after their suggestions are ignored by MDs consult their fellow nurses in the department or outside the department to confirm whether the MDs are right or wrong:

Soon after the MD reject my precise opinion about the patient's treatment, I consult with other nurses within my department regarding the opinion I gave. (P1, RN)

Some nurses reported that they do not give up easily after the MDs reject their suggestions especially if they are confident that their suggestions would result in better patient's outcome:

I take my time to explain to MDs with rationale and sometimes present to them books as a reference of my opinion. (P1, RN)

Most nurses reported that if they have absolute confidence that their suggestions would yield better patient's outcome, but had been rejected by MDs, they take appropriate steps in reporting MDs to the management of the hospital.

### 3.5 Two examples of clinical cases reported by nurses

One day, a pregnant mother with a previous uterus scar came to our hospital. She presented with labour pain. According to our policy, women with the previous scar must not deliver by spontaneous vaginal delivery (SVD) rather should undergo caesarean section (C/S). I advised the MD to allow the patient to go for C/S but the MD did not consider my opinion. Rather, he decided to allow the mother to deliver by SVD. I decided to follow the MD's decision. Meanwhile, at the second stage of labour, the baby's head had already descended but there were no contractions and at the same time, blood through vagina was observed. I reported to the MD the signs presented by the patient but he still insisted on SVD, he ordered to administer Oxytocin to induce contractions. After some time, the amount of blood through the vagina increased. Again, I reported to the MD. The MD, after he realized, that the situation was getting worse, he decided to take the patient through C/S. Unfortunately, during the procedure, it was found that the baby had already died and the mother's uterus ruptured. I felt bad and decided to report the situation to the hospital Director for further action. (P4, RN)

A pregnant woman admitted into our hospital with pregnancy discomfort. After assessed the fetal heart rates, I realized the presence of fetal distress. I spoke to the MD on duty about this finding, but he did not trust me. Rather, he decided to assess heart rates by himself and said it was normal. After few hours, it became apparent that the fetus had died. I felt bad to see a mother missing a baby because of MD's malpractice. I decided to report the concern during a departmental meeting held the next morning. Later, the MD realized I was right and accorded me with respect henceforth. Till date, I'm one of the first person he hold discussion with whenever he encounters any problem during patient's treatment. (P7, RN)

### 3.6 Factors influencing MDs to reject nurse's advice

MDs understand that nurses are professionals only to receive MD's order, but not to be involved in decision making about patient's treatment. (P1, RN)

MDs don't respect nurses and feel a nurse can not assist them to make a decision concerning patient treatment. (P2, RN)

Nurses are very important in clinical decision and understand patients better than MDs because they spend more time with patients. Always i listen to nurse's opinion and judge whether I need to follow or not. (P6, MD)

Many respondents identified educational background as an influencing factor for MDs to the rejection of their suggestions in the treatment of patients:

MDs feel they are well educated than nurses and understand everything. (P4, RN)

MDs may reject nurse's advice not because the advice is not good but rather because a nurse's level of education is comparatively low. (P1, RN)

If the MD understands daily clinical performances of a nurse, influences them to accept or reject nurse's suggestions. (P8, RN)

Many respondents said that MDs reject the suggestions of nurses just to keep to their standards as MDs not because they are of no use:

MDs do not want to be marked inferiors that is why they can do anything to maintain their status regardless of how much will affect patients. (P7, RN)

Some participants identified the stressful duties in the work of the MDs as a reason for rejecting nurse's opinion:

Sometimes MDs get tired due to many hospital activities. It becomes hard for them to discuss with nurses about patients treatments. (P2, RN)

In my department, we are only two doctors with many patients. I don't have enough time to hear nurses opinion about patient's treatment because many patient are waiting to be attended. (P10, MD)

I wish I can have time to listen the opinions from nurses concerning patient's treatment before my final decision but it is impossible because have many patients to treat. (P12, MD)

Some participants described that traditionally MDs have greater authorities in clinical decisions in the treatment of patient:

A nurse might have good opinion or something to correct but because he/she believe MDs are everything decides to remain silent. (P1, RN)

Nurses are free to provide their own opinion regarding patient's treatment, but I have power to give final decision. (P9, MD)

One healthcare provider said sometimes nurses provide suggestions that are not based on scientific facts:

I do not follow nurse's advice once it is given just from her/his experience rather must be evidence‐based suggestions. Anything that is in contrary to my knowledge of medicine I don't agree. (P6, MD)

Nurses in my department are of good help, they always provide me with scientific advice concerning patient's treatment and this has helped more patients to have a quick recovery. (P11, MD)

### 3.7 Impacts of doctor‐rejecting nurse's advice

Most of them reported the loose of enthusiasm to work after their suggestions are ignored by MDs:

When an MD rejected my opinion, I felt disrespected and the peace I had at the working place disappeared. (P3, EN)

I always talk to MDs to know why my opinion has been rejected. If the reason given is not convincing I become emotionally stressed. (P1, RN)

Many healthcare providers reported that the rejection of opinion of nurses by MDs could result in a diminished relationship between the two professionals:

Collaboration of nurses and MDs in providing health care services is affected once the precise opinions from nurses are not considered. The confidence of nurses in providing care is diminished and feels inferior. (P1, RN)

It was mentioned that the impact of disagreement in clinical decision between nurses and MDs cannot be confined at the professional level; rather, it extends further to patients and the community.

Patient may receive suboptimal treatment because of nurse‐doctor disagreements in clinical decision. (P1, RN)

The patient receives under or overdose, which may further complicate the condition of the patient. (P2, RN)

Most healthcare providers said when MDs reject nurse's suggestions, it might affect the nurse's performance:

The work motivation for nurses diminishes when they find their opinions are not appreciated by MD. (P5, EN)

Silence among nurses is because they understand that even if they speak their suggestions will not be accepted, therefore they choose to remain silent. (P4, RN)

If the MD has shouted in front of patients, those patients will absolutely feel that nurses are incompetent in their practices and more acknowledge the works of MDs. (P7, RN)

The effect may extend further to the community, as the patient can report the situation to relatives and the community as well, which may affect the reputation of the hospital and staff:

The number of patients coming to hospital having nurse‐doctors disagreement in clinical decision decreases that directly affects hospitals business. (P3, EN)

## 4 DISCUSSION

Most respondents reported that nurses must be allowed to participate in clinical decisions on the treatment of a patient. This is consistent with the nurse's guiding principle, which encourages nurses to collaborate with other healthcare providers as members of a team (TNMC, 2015). This study showed nurses' suggestions as important in a patient's recovery, which is opining to an already existing literature (Kvande, Lykkeslet, & Lisa, 2017).

### 4.1 Actions taken by nurses when doctors reject their opinion

Some nurses confidently approach MDs to enquire about the rationale of rejecting their opinions. Others sought colleague views on why their opinions were rejected by the MDs. Other nurses joined in one voice to ensure their opinions were adhered to when they feel they are right. This is similar to another study where nurses joined hands and with one voice denounced the habit of an MD by writing a letter to the administration of a particular health facility to express their discomfort in working with an MD (Maddineshat, Rosenstein, Akaberi, & Tabatabaeichehr, 2016).

Many nurses in different healthcare facilities are carrying out various tasks as part of an indirect reaction on the rejection of their opinions. For example, a nurse just goes ahead to administer a drug on a patient once s/he believes it is the right medication and the MD has refused to accept their opinion. This conforms to another study that reported same because nurses were tired of their opinions been ignored. Nurses added or reduced the dosages of medications contrary to the orders given by MDs and sometimes avoided administering the medications outright (Foth et al., 2015).

### 4.2 Factors influencing doctors rejecting nurses' opinion

Healthcare providers reported the existing traditional belief that MDs perceive themselves as superior to nurses and try to keep clinical decision‐making to themselves. Again, other studies revealed that MDs perceive themselves to be more powerful and competent than nurses (Achilles, 2010; Fagin & Garelick, 2004; Hoffman et al., 2004; Krogstad, Hofoss, & Hjortdahl, 2004). Another study also reported that MDs perceived nurses as their assistants (Elsous et al., 2017). These findings are contrary to the document prepared by Tanzania nursing and midwifery council, which shows that nursing is an independent profession and self‐regulated to function in a broader setting (TNMC, 2014).

Nurses are ignored in decision‐making because they are regarded to have no authority (Johnson, 2009; Murata, 2014) and are considered as tools for carrying out MD's orders (Fagin & Garelick, 2004). However, the nursing council exposes that nurses are advocates of individual patients, families and community to ensure safe delivery of care (TNMC, 2014). The term “advocacy” refers to nurses raising their voice on behalf of their clients, protecting patient's rights and increasing awareness for patients to understand their rights; if nurses cannot advocate for patients, then the real purpose of nursing care is diminished (Tomaschewski‐barlem, Lunardi, Luiz, Barlem, & Marcelino, 2016; Vaartio, 2008). The impression of poor educational background of nurses by MDs is also in line with another study where MDs only acknowledged nurses who were experts in wound management and yet regarded that as a simple task (Foth et al., 2015). However, studies have reported that nurses are competent in their various areas of training and are knowledgeable and skilful for patient holistic care (Kvande et al., 2017; TNMC, 2014). In addition, the guiding principles on medical ethics for (doctors) show that MDs should cooperate fully with other healthcare providers including nurses without undermining their reputation through unjustifiably criticisms (MAT, 1995).

Nurses, who are known to be hardworking and competent in caring for patients, have their opinions likely to be accepted by MDs than nurses who are known to be incompetent in clinical practices (Johnson, 2009). Lack of time and overworking among MDs is related to MDs neglect to nurse's opinion although it is reported in healthcare facilities with fewer patients that MDs do not consider the nurses' opinions. Another study revealed that MDs do not collaborate with nurses in clinical decision because of heavy clinical workload (Tang, Zhou, Chan, & Liaw 2017).

Further, some nurses give opinions on patient's treatment based on their experiences, which is a reason for the rejection of opinions by MDs. Tangible opinions should be derived from evidence (references) and not from one's experiences. This conforms to a study where nurses drew clinical decisions primarily from the experiential knowledge that was insufficient (Thompson, 2014).

### 4.3 Impacts of doctor‐rejecting nurse's advice

One‐sided clinical decision from MDs results in patient's suboptimal healthcare services. Motivations to work become affected on the part of the nurses and healthcare facilities are affected financially because its reputation is affected, which leads to a reduction in the number of patients who uses the facility. As suggested by other studies, lack of professional collaboration in decision‐making does not only affect professionals; rather, the unpleasant outcome extends to the community at large (Johnson, 2009).

### 4.4 Limitations of the study

The study was only conducted in one region, while Tanzania has a total of 28 regions making generalization impossible. Despite the fact that the information was saturated, the sample size was also small, as large sample size in qualitative studies helps to explore new and rich information, which result in more understanding of a phenomenon (Vasileiou, Barnett, Thorpe, & Young, 2018). This study did not include non‐healthcare providers, especially patients/clients who could have confirmed the impact of nurse–doctor disagreement in clinical decision about patient's treatment.

## 5 CONCLUSION

Nurses understand patient's progress deeper compared with other healthcare providers since they stay with patients for longer periods, especially when patients are on admission. “Advocacy” is an important task to be performed by nurses to ensure the rights of patients are well protected and treatments given to patients are safe and of a standard quality. Therefore, clinical decisions concerning patient's treatment should not be done in isolation by MDs; rather, it should be made with nurses included since multidisciplinary teamwork is important. Shared clinical decision power between nurses and MDs will improve interprofessional relationship that will lead to job satisfaction and effective clinical performances.

## CONFLICT OF INTEREST

The authors declare to have no conflict of interest on any section of this paper.
